# Genetic evidence for an essential role of neuronally expressed IL-6 signal transducer gp130 in the induction and maintenance of experimentally induced mechanical hypersensitivity *in vivo *and *in vitro*

**DOI:** 10.1186/1744-8069-7-73

**Published:** 2011-09-27

**Authors:** Serena Quarta, Christian Vogl, Cristina E Constantin, Nurcan Üçeyler, Claudia Sommer, Michaela Kress

**Affiliations:** 1 Div. Physiology, DPMP, Medical University Innsbruck, Innsbruck, Austria; 2 Neurology Hospital, University Würzburg, Würzburg, Germany; 3 School of Pharmacy, University of Reading, Reading, UK; 4 Institute of Physiology, University of Freiburg, Freiburg, Germany

**Keywords:** proinflammatory cytokine, Interleukin-6, chronic pain, nociceptor sensitization, hyperalgesia, allodynia

## Abstract

Tenderness and mechanical allodynia are key symptoms of malignant tumor, inflammation and neuropathy. The proinflammatory cytokine interleukin-6 (IL-6) is causally involved in all three pathologies. IL-6 not only regulates innate immunity and inflammation but also causes nociceptor sensitization and hyperalgesia. In general and in most cell types including immune cells and sensory neurons, IL-6 binds soluble μ receptor subunits which heteromerizes with membrane bound IL-6 signal transducer gp130. In the present study, we used a conditional knock-out strategy to investigate the importance of signal transducer gp130 expressed in C nociceptors for the generation and maintenance of mechanical hypersensitivity. Nociceptors were sensitized to mechanical stimuli by experimental tumor and this nociceptor sensitization was preserved at later stages of the pathology in control mice. However, in mice with a conditional deletion of gp130 in Nav1.8 expressing nociceptors mechanical hypersensitivity by experimental tumor, nerve injury or inflammation recovery was not preserved in the maintenance phase and nociceptors exhibited normal mechanical thresholds comparable to untreated mice. Together, the results argue for IL-6 signal transducer gp130 as an essential prerequisite in nociceptors for long-term mechanical hypersensitivity associated with cancer, inflammation and nerve injury.

## Background

Tenderness, hypersensitivity to mechanical stimulation and pain are classical symptoms of inflammation and reduce the quality of life in particular in patients suffering from arthritis but also malignant tumor and neuropathy. The classical proinflammatory cytokine interleukin-6 (IL-6) is produced and excreted by immune cells including macrophages, glia cells and even neurons (reviewed in [1]. IL-6 plays a major role in the pathogenesis of rheumatoid arthritis (RA). Elevated levels of IL-6 can be detected in serum and synovial fluid of RA patients and correlate with disease activity [2,3]. Some types of tumors produce IL-6 [4], for example, elevation of serum IL-6 levels is found in up to 60% of lung cancer patients in advanced stages [5]. Following nerve injury elevated IL-6 levels correlate well with development of thermal hyperalgesia and mechanical hypersensitivity (allodynia) [6-8]. Due to its importance in controlling innate immunity and inflammation, IL-6 is generally accepted to contribute to pain and hypersensitivity associated with inflammation, neuropathy or cancer. IL-6 induces heat hypersensitivity both *in vitro *and *in vivo*, which is mediated by regulation of TRPV1 [9-12]. Mice carrying a null mutation of IL-6 develop less thermal hyperalgesia after experimental inflammation or nerve lesion [7,13,14], and IL-6 neutralizing antisera inhibit hyperalgesia [15].

Whereas IL-6 signal transducer gp130 is ubiquitously expressed IL-6 requires presence of a ligand binding soluble receptor (sIL-6R) subunit to induce its cellular effects. Practically all sensory neurons in the dorsal root ganglion express gp130 in the cell membrane [12,16]. IL-6/sIL-6R via gp130 induces thermal hypersensitivity both *in vitro *and *in vivo*, which is mediated by activation of PKC-δ and subsequent regulation of TRPV1 [9-12]. Conditional deletion of gp130 in Nav1.8 expressing cells reveals a key role for gp130 expressed in nociceptors for cancer induced thermal hypersensitivity [10]. More importantly, IL-6 induces mechanical hypersensitivity and triggers fast nociceptor sensitization to mechanical stimuli; co-administration of neutralizing soluble gp130 (sgp130) protein prevents IL-6 induced sensitization of C mechanonociceptors [17,18]. Since gp130 is ubiquitously expressed (for review see [19]) it cannot be decided whether the effect of IL-6 is produced by direct action of IL-6 at the nerve terminal itself or by indirect action of IL-6 on e.g. immune cells and secondary release of other neuroimmune signals [17].

Therefore, we used a conditional knock-out strategy to investigate the importance of signal transducer gp130 expressed in C nociceptors for the generation and maintenance of mechanical hypersensitivity in three mouse models of pathological and persistent pain. We analyzed von Frey mechanical sensitivity *in vivo *and performed single fiber recordings *in vitro*. Our data provide significant evidence for long lasting mechanical hypersensitivity *in vivo *and nociceptor sensitization *in vitro *in control mice following experimental cancer, inflammation or neuropathy. Mice with a null mutation of gp130 in Nav1.8 expressing nociceptive primary afferents (SNS-gp130^-/-^) initially showed signs of nociceptor sensitization and hypersensitivity to mechanical stimuli which, however, were not as prominent as in the control mice. Moreover, mechanical hypersensitivity in SNS-gp130^-/- ^mice recovered in the maintenance phase in all three models of pathological pain. This significant benefit of gp130 deletion in Nav1.8 expressing nociceptors suggests that gp130 signal transducer is a direct and important regulator of mechanical hypersensitivity in particular in the maintenance phase of chronic pain models.

## Results

### Role of gp130 expressed in nociceptive primary afferents for tumor-induced mechanical hypersensitivity

**Figure 1:**
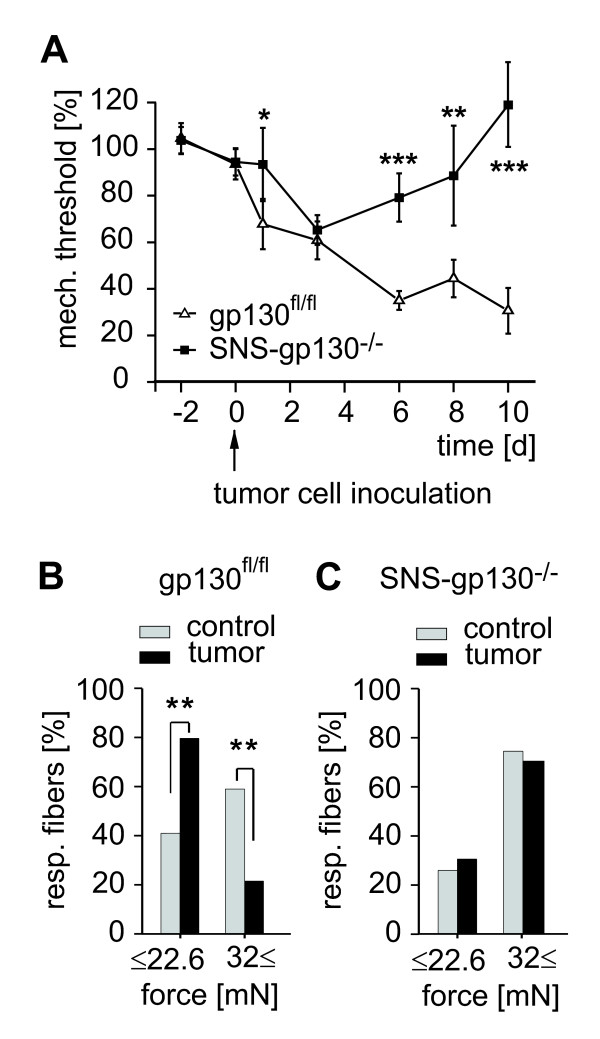
**Neuronal signal transducer gp130 plays a role in tumor-induced mechanical hyperalgesia**. **(A) **Mechanical threshold after tumor induction in the hind-paw of gp130^fl/fl ^(open triangle n = 17) and SNS-gp130^-/- ^(black square, n = 11) mice. After inoculation with tumor cells mechanical thresholds dropped by 30% in both mouse strains, but in SNS-gp130^-/- ^mice the degree of hypersensitivity was significantly reduced in comparison to gp130^fl/fl ^mice at day 1, 6, 8, and 10 (* p < 0.05, ** p < 0.01, *** p < 0.001; ANOVA). **(B, C) **Mechanical von Frey thresholds of fibers recorded in *in vitro *skin-nerve preparation from gp130^fl/fl ^and SNS-gp130^-/- ^mice at 7 to 10 days post inoculation. Nociceptors from gp130^fl/fl ^mice projecting into tumor area showed lower thresholds (≤ 22.6 mN, n = 28) than fibers innervating healthy skin (n = 66, ** p < 0.01; χ^2^-test), whereas no difference was found between healthy and tumor skin in SNS-gp130^-/- ^animals.

In gp130^fl/fl ^and SNS-gp130^-/- ^mice, experimental tumors as assessed from the tumor size at 10 days after tumor cell inoculation were similar in both groups (data not shown). Tumor growth was accompanied by increasing mechanical hypersensitivity and a decrease of mechanical withdrawal thresholds to 68.4 ± 10.9% of control values in gp130^fl/fl ^mice (n = 17). On the other hand, at the first day after inoculation mechanical sensitivity was not significantly changed in SNS-gp130^-/- ^mice (SNS-gp130^-/-^, n = 11, 94.2 ± 15.9% in comparison to gp130^fl/fl^, n = 17, 68.4 ± 10.9%, p < 0.05; two way RM ANOVA, Tukey post-test; genotype: F(1, 166) = 15.376; p < 0.001, time points: F(6, 166) = 7.739; p < 0.001, genotype × time points: F(6, 166) = 3.372; p < 0.01; Figure 1A). Mechanical thresholds dropped by 30% within the first three days in SNS-gp130^-/- ^mice but mechanical hypersensitivity significantly recovered from day 6 after tumor cell inoculation (SNS-gp130^-/-^, n = 11, 79.9 ± 10.4% in comparison to gp130^fl/fl^, n = 17, 35.4 ± 4.0%, p < 0.001; ANOVA). Differences between the two groups became even more evident at later time points.

As a possible mechanism two general possibilities are plausible. It is generally accepted that increased efficiency of spinal synaptic transmission is a major mechanism of mechanical hyperalgesia and allodynia. Alternatively, sensitization of primary nociceptive afferents could occur. To determine whether peripheral nociceptor sensitivity to mechanical stimuli was affected we performed standard teased fiber recordings from nociceptors at 7 to 10 days post inoculation, *in vitro*. Nociceptors with receptive fields within the tumor region had significantly lower mechanical von Frey thresholds than fibers innervating healthy skin in gp130^fl/fl ^mice (untreated: median: 32 mN, 17.65 mN and 83.5 mN as upper and lower quartile, n = 66 vs. tumor: median 16 mN, 11.4 mN and 22.6 mN as lower and upper quartile, n = 28). In healthy skin, 41% of mechanosensitive fibers responded to mechanical stimuli equal to 22.6 mN or lower whereas 59% were sensitive to 32 mN or higher (n = 66). In tumor associated skin, a significantly larger percentage of fibers (78%) responded to von Frey mechanical stimulation with less than 22.6 mN (n = 28, p < 0.01; χ^2^-test, Figure 1B). In contrast, mechanical sensitivity was similar of nociceptors projecting into healthy skin (n = 35) or tumor skin in SNS-gp130^-/- ^mice (n = 20; n.s.; χ^2^-test, Figure 1C, E). These results suggest that the signal transducer gp130 expressed is causally involved in tumor-associated mechanical hypersensitivity of nociceptors.

### Reduced mechanical hypersensitivity of SNS-gp130^-/- ^mice in neuropathic and inflammatory pain models

**Figure 2:**
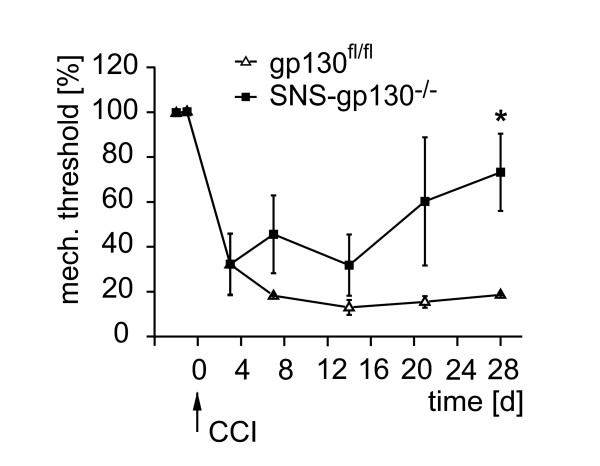
**Mechanical hyperalgesia in neuropathic pain model required neuronal gp130**. After chronic constriction injury (CCI), SNS-gp130^-/- ^mice (black square, n = 14) showed significant recovery in comparison to gp130^fl/fl ^mice (open triangle, n = 13, * p < 0.05; *** p < 0.001; ANOVA and post-hoc Tukey test).

Although cancer pain appears to be unique and distinct from other chronic pain states [20] it seems to share at least some characteristics associated with inflammation [21] and also neuropathy [22]. Therefore, we aimed to address whether gp130 plays a role in regulating mechanical hypersensitivity in other persistent pain models. In the CCI model of neuropathic pain, both, gp130^fl/fl ^and SNS-gp130^-/- ^mice consistently developed a significant decrease of mechanical thresholds after nerve lesion. While control mice remained hypersensitive throughout the entire observation period, SNS-gp130^-/- ^mice partially recovered from the hypersensitive stated (day 28: percent decrease related to base line threshold gp130^fl/fl ^23.9 ± 1.9%, n = 13, vs. SNS-gp130^-/- ^67.2 ± 10.8%, n = 14; p < 0.05, p < 0.001; two way RM ANOVA, Tukey post-test; genotype: F(1, 181) = 4.820; p < 0.05, time points: F(6, 181) = 28.333; p = < 0.001, genotype × time points: F(6, 181) = 2.257; p < 0.05; Figure 2). This suggests that gp130 is also involved in the maintenance of neuropathic mechanical hypersensitivity.

**Figure 3:**
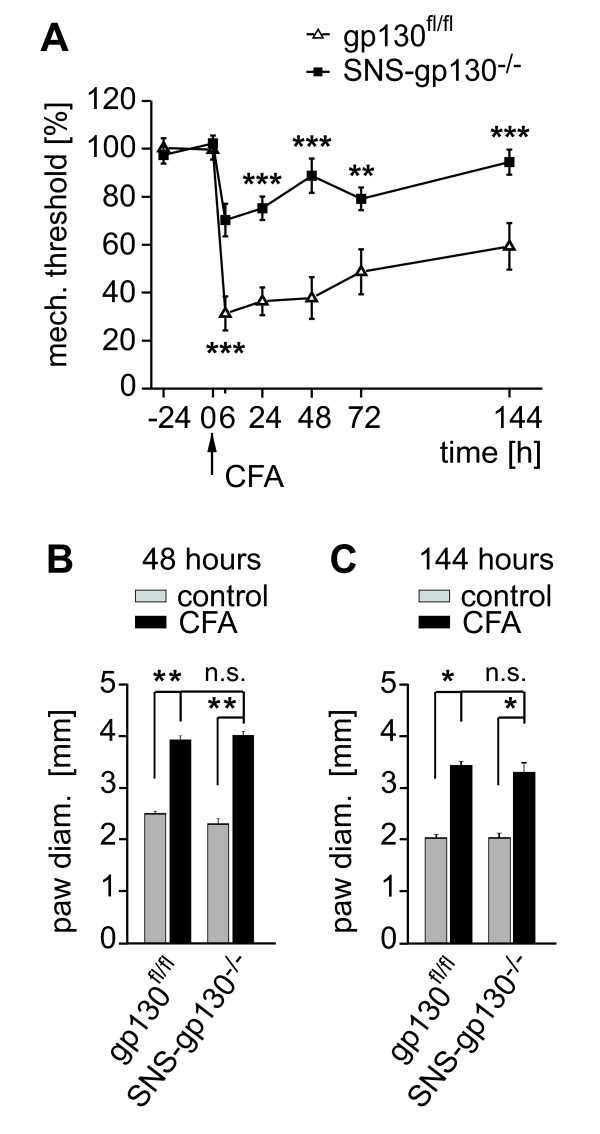
**Lack of signal transducer gp130 reduced inflammation-induced mechanical hyperalgesia**. **(A) **Unilateral hind-paw injection of CFA induced a drop of mechanical threshold after 6 h in both SNS-gp130^-/- ^mice (black square, n = 8) and gp130^fl/fl ^mice (open triangle, n = 8). SNS-gp130^-/- ^mice showed attenuated mechanical hypersensitivity at all the time points tested after injection compared to gp130^fl/fl ^mice (** p < 0.01, *** p < 0.001; ANOVA). **(B, C) **Similar degree of paw swelling in both mouse strains at 48 and 144 h after CFA injection (* p < 0.05, ** p < 0.01; Mann-Whitney U-test).

Likewise, mechanical hypersensitivity is generally observed after subcutaneous injection of CFA. In gp130^fl/fl ^mice, subcutaneous injection (1 mg/ml) into the plantar site of the hind-paw resulted in paw swelling and a drop of mechanical von Frey thresholds to 31.3 ± 7.1% after 6 hours. The CFA-induced mechanical hypersensitivity was significantly attenuated in SNS-gp130^-/- ^mice (70.3 ± 6.8%, n = 8, p < 0.001; two way RM ANOVA, Tukey post-test; genotype: F(1, 104) = 21.439; p < 0.001, time points: F(6, 104) = 19.368; p < 0.001, genotype × time points: F(6, 104) = 5.165; p < 0.001; Figure 3A). Furthermore, control animals maintained the dramatic reduction of mechanical thresholds at 48 hours while SNS-gp130^-/- ^mice were largely resistant to mechanical hypersensitivity also in the maintenance phase of CFA induced inflammation (SNS-gp130^-/- ^88.8 ± 7.1% vs. gp130^fl/fl ^37.8 ± 8.7%, n = 8, p < 0.001; ANOVA). Six days after injection mechanical thresholds of SNS-gp130^-/- ^mice returned almost to baseline values whereas control mice were still mechanically hypersensitive (SNS-gp130^-/- ^94.5 ± 5.2% vs. gp130^fl/fl ^59.3 ± 9.7%, n = 8, p < 0.001; ANOVA). This difference could not be simply explained by a major reduction of inflammation, since at least the degree of swelling was similar in both mouse strains at 48 and 144 hours (Figure 3B, C).

Taken together, the data from the behavioral analysis of the cancer pain and the CFA inflammatory pain models in combination with *in vitro *recordings from unmyelinated nociceptive afferents, suggest that signal transducer gp130 expressed in peripheral nociceptors is critical for mechanical hypersensitivity and nociceptor sensitization during both induction and maintenance phases. Cancer pain shares certain aspects of inflammatory as well as neuropathic pain. A recovery from mechanical hypersensitivity was also found in the CCI model for neuropathic pain. This argues for a more general role of gp130 expressed in nociceptors not only for the generation but also for the maintenance of mechanical hypersensitivity independent of the underlying disease.

## Discussion

In the present study we have shown for the first time that the IL-6 signal transducer gp130 in Nav1.8 expressing primary afferents has little impact on the induction of mechanical hypersensitivity but is critically involved in the maintenance of nociceptor sensitization to mechanical stimuli in a mouse model of cancer pain. Mice lacking gp130 in nociceptors showed some signs of mechanical hypersensitivity during the first days after induction of experimental malignant soft tissue cancer that were comparable to controls. The mice significantly recovered from hypersensitivity in the later stages of the observation period. In contrast, mechanical hypersensitivity progressively became more severe in gp130 expressing control animals. The delayed recovery of mechanical hypersensitivity suggests a critical role of gp130 dependent signaling not only for the induction but more prominently for the maintenance of long-term mechanical hypersensitivity in cutaneous nociceptors. Cancer pain is considered exceptional and at least partially distinct from neuropathic and inflammatory pain. However, our data show that gp130 expressed in nociceptors is also essential for the development of mechanical hypersensitivity following inflammation and/or nerve injury. Together, the data suggest that IL-6 signal transducer gp130 is an essential prerequisite for long-term mechanical hypersensitivity associated with cancer, inflammation and nerve injury.

In humans all three mentioned conditions are characterized by pronounced mechanical hyperalgesia and/or allodynia and in mouse models corresponding hypersensitivity to noxious and/or innocuous mechanical stimuli is regularly reported. Although the pathologies are complex and specific for the respective disease, they share certain aspects of inflammatory reactions involving components of innate immunity including sequential release of cytokines [23]. In particular, cytokines of the IL-6 family are important regulators of the immune response. There is increasing evidence that IL-6 like cytokines may be causally involved in the etiology of neuropathic pain and mechanical allodynia following malignant tumor, nerve injury or inflammation [24]. The IL-6 like cytokine family includes IL-11, IL-27, leukaemia inhibitory factor (LIF), ciliary neurotrophic factor (CNTF), oncostatin M (OSM), cardiotrophin (CT-1), neuropoietin, cardiotrophin-like cytokine (CLC) and B cell stimulating factor (BSF-3). The question arises which of these members is most critical for the regulation of nociceptor sensitivity. Although not considered a classical proinflammatory cytokine, LIF appears to be an interesting candidate since LIF mRNA is up-regulated in inflammation [25]. LIF receptor is expressed in DRG neurons and up-regulated by nerve injury [16]. However, the role of LIF in nociception is still controversially discussed. Although LIF differentially regulates capsaicin and heat sensitivity in cultured sensory neurons [26], LIF injection into the mouse paw induces local mechanical, but not thermal hypersensitivity [10,27]. In the CFA inflammation model LIF has anti-inflammatory and analgesic effects [28]. In addition, OSM has certain roles in inflammation [29]. OSM receptors are expressed in DRG neurons, are associated with nociceptor sensitization in inflammation and form heterodimers with gp130 [30-32].

Although several members of the IL-6 family have been associated with painful conditions, research in the last years has mainly focused on IL-6. Increased IL-6 serum levels have been detected in patients with neuropathy, malignant tumors, musculoskeletal disorders, burn injury or autoimmune and chronic inflammatory conditions like RA [33-38]. IL-6 is up-regulated following experimental peripheral nerve injury and exhibits a growth promoting effect on primary sensory neurons [39-42]. Intraplantar, intracerebroventricular or intrathecal injection of IL-6 induces thermal and mechanical hypersensitivity in rodents [6,10,17,18,43,44]. In addition, recordings from nociceptors *in vivo *and *in vitro *revealed a role for IL-6 in sensitizing nociceptors to thermal and mechanical stimulation [11,12,17]. IL-6-/- mice show a phenotype with reduced thermal hypersensitivity after experimental inflammation or nerve constriction [7,13,14]. Antisera neutralizing endogenous IL-6 inhibit inflammatory hyperalgesia [15] and the orally available, small molecule IL-6 receptor antagonist TB-2-081 reverses pain in a pancreatitis rodent model [45]. Moreover, neutralizing IL-6 strategy has evolved as effective pain therapy in humans [46].

Most cytokines of the IL-6 family bind to heteromeric complexes composed of ubiquitously expressed gp130 and distinct μ receptor subunits with signal transduction domains (e.g. LIF-R or OSM-R). In contrast, IL-6 signaling entirely depends on the availability of gp130 homomers which are activated by IL-6 bound to the ligand binding IL-6 receptor μ-subunit (IL-6-R) which is present in few cell types only [47,48]. In most systems including sympathetic neurons, IL-6 effects depend on the presence of the soluble IL-6 receptor (sIL-6R) [49] which after ligand binding heteromerizes with membrane bound gp130 [47,50]. Furthermore, IL-6/sIL-6R complex or Hyper-IL-6 (HIL-6), a synthetic fusion protein mimicking the IL-6/sIL-6R complex [51,52], increase nociceptor responsiveness and induce thermal hypersensitivity [9,11,12]. A dual regulation of heat sensitivity by IL-6 and its soluble receptor sIL-6R has been reported [11]. The sensitization involves activation of Janus tyrosine kinase (JAK), adapter proteins Gab1 and Gab2 and ultimately PKC-δ which regulates the heat transducer ion channel TRPV1 [10,12]. Despite a recent report that IL-6 but not the signal transducer gp130 is up-regulated in neuropathic rats [53] gp130 seems to play a crucial role in pathological pain since antagonizing sgp130 prevents acute nociceptor sensitization in experimental arthritis [17]. This acute effect of IL-6 on mechanosensitivity in this study rather seems to be partially indirect. We have previously reported that gp130 expressed in nociceptors is required for IL-6 induced regulation of TRPV1 and thermal hypersensitivity [10]. Here we show that mice lacking gp130 in nociceptors (SNS-gp130^-/-^) develop but recover from mechanical hypersensitivity in mouse models of cancer, inflammatory and neuropathic pain. Our data suggest that gp130 expressed in nociceptors is a critical regulator of the maintenance of mechanical hypersensitivity in nociceptors in particular in the CCI mouse model for neuropathic pain.

At least three possible signaling pathways may be activated following gp130 activation: the classical signal transducer and activator of transcription 3 (STAT3) pathway is activated in primary afferent neurons by peripheral inflammation possibly through OSM receptor [32]. Although STAT3 signaling is beneficial to axonal growth through activating transcription of unidentified genes in DRG neurons [54], STAT3 is differentially activated by IL-6 cytokines in DRG sensory neurons by CNTF and LIF but not IL-6 [55]. For thermal hypersensitivity IL-6 signals via activation of the adapter proteins Gab1/2, PI_3_K, PKC-δ and regulation of TRPV1 [10]. In contrast, the sequelae of mechanical hypersensitivity and even mechanical nociceptive transduction remain largely enigmatic to date. Recently, the importance of translation for the regulation of de novo protein synthesis has been discovered for IL-6 induced mechanical nociceptive plasticity which is blocked by inhibitors of general and cap-dependent protein synthesis [18]. Although Mnk1 and ERK have been reported as upstream regulators of the translation factor eIF4F [18], the nature of possible downstream target proteins accounting for IL-6 mechanical hypersensitivity remain to be elucidated. In Purkinje neurons, chronic IL-6 exposure alters electrophysiological properties and calcium signaling [56]. Such general increases in excitability may account for mechanical nociceptive plasticity. Nonetheless, IL-6 has not been found to enhance excitability in unmyelinated sensory axons in normal and injured peripheral nerve [57]. Therefore more likely, ion channels potentially involved in mechanotransduction may be regulated by gp130 dependent translation or transcription [58,59]. Although a direct link of IL-6 and such channels is still absent, the up-regulation of IL-6 and the mechanosensitive ion channel TRPA1 in mustard oil colitis [60] may be indicative for the regulation of TRP and other mechanosensitive ion channels by IL-6/gp130 signaling. Further studies will be required to elucidate the final target of gp130 in mechanonociception.

## Conclusions

In the present study, we reveal that gp130 expressed in nociceptors is a key regulator of mechanical hypersensitivity in the induction and even more in the maintenance phase of three major pathologies associated with severe and long-lasting hypersensitivity and pain. Our results strongly support a critical role for gp130 in nociceptive primary afferents as a chronification factor. On the basis of our results, the launch of inhibitors for IL-6 or gp130 as a novel class of anti-inflammatory drugs should not only give rise to great hopes for the treatment of inflammation in rheumatoid arthritis [61-63] but also for alleviation of sustained pain as the symptom that most severely reduces the patients' quality of life.

## Methods

### Animals

Male C57Bl6 mice (> 8 weeks old) were used in all experiments. Mice were housed on a 12 h light/dark cycle with free access to chow and water and all animal use procedures were in accordance with ethical guidelines and animal welfare standards according to Austrian law. Mice were assigned to the following experimental groups: group I did not receive any treatment and was used as control; group II was inoculated with tumor cells; group III was injected intraplantarly with CFA; group IV underwent surgical procedure of chronic constriction injury (CCI). All groups contained gp130^fl/fl ^control and SNS-gp130^-/- ^mice, generated by gene targeting as described previously [10].

### Behavioral tests

Standard testing procedures were used to quantify signs of pain-like behavior reflected in changes in mechanical sensitivity following inflammation, nerve lesion and tumor development. The area tested was the plantar side of the hind-paw where the tumor cells or CFA were inoculated. Baseline measurements were taken 2 times before treatment and daily thereafter up to 10 days post inoculation. Mice were placed in a plastic chamber with a wire mesh floor and allowed to habituate for 1 h before starting the test. Mechanical sensitivity at the site of tumor cells implantation was determined by measuring the paw withdrawal threshold in response to probing of the plantar surface of the hind-paw with calibrated von Frey monofilaments with bending forces between 2.8 and 45.3 mN. The withdrawal threshold was determined by increasing and decreasing stimulus intensity on the basis of the up-down method [64,65], where a 11.4 mN stimulus was applied first. Behavioral test were performed in accordance with ethical guidelines and Austrian law. All measurements were done blindly.

### Skin-nerve preparation and single fiber recordings

An *in vitro *skin nerve preparation [10,21,66] was used to investigate the properties of unmyelinated afferent nerve fibers innervating the skin in the tumor area. In mice from group II electrophysiological recordings were performed 7 to 10 days post inoculation when a tumor mass had developed at the injection site covering the saphenous nerve territory. Animals were killed by CO_2 _inhalation. The saphenous nerve was dissected with the skin of the dorsal hind-paw attached and mounted in an organ bath "inside-up" to expose the corium side. The preparation was superfused (15 ml/min) with an oxygen-saturated modified synthetic interstitial fluid solution containing (in mM) 108 NaCl, 3.48 KCl, 3.5 MgSO_4_, 26 NaHCO_3_, 1.7 NaH_2_PO_4_, 2.0 CaCl_2_, 9.6 sodium gluconate, 5.5 glucose, 7.6 sucrose at temperature of 31 ± 1°C and pH 7.4 ± 0.05 [67]. The saphenous nerve was pulled into a separate chamber of the organ bath and placed on a small mirror. With the use of sharpened watchmakers' forceps fine filaments were teased from the desheathed nerve and placed on a gold wire recording electrode. Action potentials in single sensory neurons were recorded extracellularly, amplified (5000×), filtered (low pass 1 kHz, high pass 100 Hz), visualized on an oscilloscope and stored on a PC-type computer with Spike/Spidi software package for offline analysis employing a template-matching procedure [10,12,21]. The fibers were characterized as unmyelinated (C) according to their conduction velocity (< 2 m/s, calculated from the latency of unitary action potential to electrical stimulus at receptive field and distance of receptive field to recording electrode) and on the basis of the shape of the action potential. The receptive field of the primary afferent fiber was located by mechanical probing of the skin with a blunt glass rod. Only units with a signal-to-noise ratio > 2 were used for further analysis. Fibers were subject to a standard protocol of adequate mechanical and thermal stimuli. The mechanical threshold of each unit was determined with a set of calibrated von Frey monofilaments with uniform tip diameter 1.1 mm and bending forces ranging from 1 to 362 mN. The strength of the finest filament which evoked at least 3 action potentials was defined as activation threshold.

### Tumor cell culture and implantation

Lung carcinoma cells (ATCC clone 1642, American Type Cell Culture Collection) were cultivated on 25 cm^2 ^flasks in Dulbecco's modified Eagle's medium (DMEM, PAA, Vienna, Austria) with 4 mM L-glutamine and 10% fetal bovine serum (FBS), were grown to confluence, fed and passed once a week. Tumor cells were prepared for implantation by pouring off the media and rinsing with phosphate buffer saline (PBS). Trypsin-EDTA (0.5%, 1×, Gibco, Austria) was added for 2 min to detach cells from the flask. The enzymatic reaction was quenched by addition of modified DMEM. Just prior to implantation cells were counted, washed twice and then re-suspended in PBS for implantation. Mice were anaesthetized with isoflurane (Baxter, Vienna) and 7 × 10^5 ^lung carcinoma cells in a volume of 25 μl PBS were injected subcutaneously in the plantar and dorsal site of the mouse hind-paw.

### CFA drug preparation and administration

Complete Freud's Adjuvant (CFA, Sigma) was injected intracutaneously in a total volume of 25 μl and animals of group III were tested for mechanical sensitivity at 6, 24, 48, 72 and 144 h after injection. At 48 hours and the terminal day of the behavioral test, the vertical foot diameter of the injected and the non-injected paw was determined using a caliper.

### Chronic constriction injury

The CCI model was obtained by three ligatures (7-0 prolene) on the right sciatic nerve with a distance of 1 mm each [68,69]. The ligatures were tied around the nerve proximal to the trifurcation until a short flick of the hind-paw. Mice were anesthetized by intraperitoneal Phenobarbital injection before the procedure.

### Statistics

For statistical analysis the SigmaStat 3 software was used. Data are presented as mean ± SEM if not otherwise state. Two-way repeated measure ANOVA followed by Tukey post-hoc test or Mann-Whitney U-test for comparison between groups were used. For comparison of relative group sizes χ^2^-test was calculated. Differences were considered statistically significant at p < 0.05.

## Competing interests

The authors declare that they have no competing interests.

## Authors' contributions

SQ, CV and NÜ carried out behavioural experiments. CV and CEC performed electrophysiological recordings from single fibers in vitro, CS and MK participated in the design of the study and coordination, SQ and MK drafted the manuscript. All authors read and approved the final manuscript.
